# A chromosome-level genome of electric catfish (*Malapterurus electricus*) provided new insights into order Siluriformes evolution

**DOI:** 10.1007/s42995-023-00197-8

**Published:** 2023-12-14

**Authors:** Meiru Liu, Yue Song, Suyu Zhang, Lili Yu, Zengbao Yuan, Hengjia Yang, Mengqi Zhang, Zhuocheng Zhou, Inge Seim, Shanshan Liu, Guangyi Fan, Huanming Yang

**Affiliations:** 1 College of Life Sciences, University of Chinese Academy of Sciences, Beijing, 100049 China; 2 BGI-Qingdao, BGI-Shenzhen, Qingdao, 266555 China; 3 BGI-Shenzhen, Shenzhen, 518083 China; 4 Professional Committee of Native Aquatic Organisms and Water Ecosystem of China Fisheries Association, Beijing, 100125 China; 5 Integrative Biology Laboratory, College of Life Sciences, Nanjing Normal University, Nanjing, 210023 China; 6 State Key Laboratory of Agricultural Genomics, BGI-Shenzhen, Shenzhen, 518083 China

**Keywords:** Electric fish, Karyotype analysis, Convergent evolution, Environmental adaptation, Historical effective population size

## Abstract

The electric catfish (*Malapterurus electricus*), belonging to the family Malapteruridae, order Siluriformes (Actinopterygii: Ostariophysi), is one of the six branches that has independently evolved electrical organs. We assembled a 796.75 Mb *M. electricus* genome and anchored 88.72% sequences into 28 chromosomes. Gene family analysis revealed 295 expanded gene families that were enriched on functions related to glutamate receptors. Convergent evolutionary analyses of electric organs among different lineage of electric fishes further revealed that the coding gene of rho guanine nucleotide exchange factor 4-like (*arhgef4*), which is associated with G-protein coupled receptor (GPCR) signaling pathway, underwent adaptive parallel evolution. Gene identification suggests visual degradation in catfishes, and an important role for taste in environmental adaptation. Our findings fill in the genomic data for a branch of electric fish and provide a relevant genetic basis for the adaptive evolution of Siluriformes.

**Supplementary Information:**

The online version contains supplementary material available at 10.1007/s42995-023-00197-8.

## Introduction

Catfish, belonging to the ray-finned fish order Siluriformes, are a diverse group of fishes that occupy various habitats and are found worldwide. With 4110 species, 498 genera, and 39 families, they make up 11.34% of all extant fishes (Fricke et al. 2021). These nocturnal creatures rely on non-visual senses, such as touch, chemically sensitive tentacles, or enlarged olfactory organs, to navigate their environments (Lundberg and Friel. 2003). Electric catfish, which are benthic and live in turbid waters, feed on benthic insects (Fagbenro et al. 2001). Compared to other sensors such as those related to taste (Bauer 1968), *Malapterurus electricus* has only a small, incomplete visual system (Ebbesson and Donnel 1980). Despite their large species number, only 22 catfish genomes are available at NCBI Genomes (Kitts et al. 2016). Here, we considered the Malapteruridae, a family where all 21 species have evolved electric organs (Fricke et al. 2021). We generated a chromosome-level genome assembly of *M. electricus*, an archetypical electric catfish species that possesses electric organs for predation and defense (Janetzko et al. 1987; Schikorski et al. 1992).

Through this analysis, we have revealed aspects of the genome and the chromosome evolution of electric catfishes, considered the phylogeny and the demographic history of *M. electricus*, and identified single genes and families of genes that may be associated with the evolution of electric organs. To explore environmental adaptations with electric catfish, we also identify and comparatively analyze visual and taste genes. Our findings may benefit future basic studies and conservation efforts of this species for further genetic and evolutionary studies of Siluriformes fish.

## Materials and methods

### DNA sample preparation and library construction

High-quality DNA for sequencing was extracted from muscle tissue using a modified DNA extraction for vertebrate tissues protocol from the tissue sample. The extracted DNA was fragmented and subjected to paired-end sequencing library construction, following single tube Long Fragment Read (stLFR) protocol (Wang et al. 2019). For Hi-C library sequencing, ~ 1 g of living muscle tissue was used for DNA extraction and library contraction, according to the method described by (Wang et al. 2019).

### Genome sequencing and assembly

Sequencing was conducted on a BGISEQ-500 sequencer, generating 118.87 Gb stLFR raw data and 307.07 Gb raw Hi-C data (Supplementary Table S1). Data filtering was then carried out using SOAPnuke software (version 1.5) (Chen et al. 2018) with the default parameters. After data filtering, 63.65 Gb of data remained for de novo assembly (the ‘clean Hi-C data’ contained 143.83 Gb). The clean reads were then pre-processed to be compatibly handled by supernova v2.0.2 (Wong et al. 2018), following the stLFR2Supernova pipeline (https://github.com/BGI-Qingdao/stlfr2supernova_pipeline). Then, Gapcloser 1.12 (Luo et al. 2012) with default parameters was used to fill gap (N) regions (Supplementary Table S2). The size of the *M. electricus* genome was estimated based on k-mer analysis (Liu et al. 2013).

The clean Hi-C data generated from Hi-C library were used to improve the connection integrity of the scaffolds (Supplementary Table S1). All valid pairs of reads were detected by mapping clean Hi-C reads to draft genome sequences using Hic-Pro v3.2 (Servant et al. 2015) and then aligned to the genome using Juicer v1.5 (Durand et al. 2016). The assembled fragments of DNA were ordered and oriented by 3D-DNA pipeline version 170123 (Dudchenko et al. 2017) based on the Juicer Hi-C contacts (“*merged_nodups.tx*t” file). Juicebox Assembly Tools v 1.11.08 (Dudchenko et al. 2017) were used for manual review and refinement to identify and remove the remaining assembly errors.

To evaluate the completeness of our assembly, the BUSCO (University of Geneva Medical School and Swiss Institute of Bioinformatics, Geneva, Switzerland; version 3.03, RRID:SCR_015008) (Manni et al. 2021; Waterhouse et al. 2018) with ray-finned fishes (actinopterygii obd10) orthologues was used to evaluate the completeness of our assembly. To assess the heterozygosity and accuracy of the assembled genome, we carried out variant calling using bcftools-1.4 (Danecek et al. 2021). The quality and barcode-trimmed stLFR data was mapped to the assembly using BWA v. 0.7.12 (Li and Durbin 2009) with default parameters, and called the SNP variant using bcftools-1.4 (Danecek et al. 2021) (mpileup parameters: *–Ou –C 30 –d 100*; call parameters: *–c –Ov*).

### Repeat annotation, gene prediction, and functional annotation

Repetitive elements in the *M. electricus* were identified using a combination of the de novo and homology-based approaches. A de novo repeat library was first construct by RepeatModeler (v1.0.11), then repeat elements were identified by RepeatMasker (v4.0.7) (Tarailo-Graovac and Chen 2009) based on the RepBase 21.01 (Bao et al. 2015) library and the de novo repeat database. Repeats on protein level were annotated by the RepeatProteinMask package in RepeatMasker based on the TE protein database, and tandem repeats were annotated by the Tandem Repeat Finder (TRF, v4.0.9) (Benson 1999). Finally, non-redundant repeats were checked according to their alignments in the genome.

We used de novo, homology-based and RNA seq data to identify protein-coding genes. After repeat masking, de novo prediction was performed using AUGUSTUS 3.0.3 (Hoff and Stanke 2019; Stanke et al. 2006) with *Danio rerio* as the HMM model species. For homology-based annotation, six homolog species (*Electrophorus electricus*, *Ictalurus punctatus*, *D. rerio*, *Lepisosteus oculatus*, *Takifugu rubripes* (GCA_901000725.2), *Rhincodon typus* (GCA_001642345.2), Supplementary Table S3), and the actinopterygii_odb9 database were aligned against the genome assembly using BLAT software version 0.36 (Kent 2002) with E-value threshold of 1e-5. The best alignments were extended 2 Kb on the both sides; then the possible gene structures were predicted using GeneWise software version 2.4.1 (Birney et al. 2004) based on the sequences. For RNA seq data, SRA data download from Sequence Read Archive database (SRA, http://www.ncbi.nlm.nih.gov/sra, Supplementary Table S4) were clipped and trimmed using SOAPfilter (version 2.2) package from SOAPdenovo2 (Luo et al. 2012) to trim five bases at the 5’ end of all reads and to discard the quality value < 20 and those reads with N bases > 10. Then the filtered reads were mapped to the assembled genome using Tophat 2.1.2 (Kim et al. 2013) with default parameters; the BAM file obtained was used for transcriptome splicing by cufflinks 2.2.1 with default parameters.

Then GeneModelMapper pipeline v.1.6.1 (GeMoMa) (Keilwagen et al. 2019) was used to integrate the gene model prediction. The homolog- and de novo-based alignments of protein-coding genes and RNA seq mapping results above were used as GeMoMa input files. Extract RNA seq evidence (ERE) and DenoiseIntrons programs were used for refining and incorporating intron boundaries according to the mapped RNA-Seq data; and GeMoMa Annotation Filter (GAF) and AnnotationFinalizer programs were used to integrate all the evidence.

The protein sequences translated from the gene structure prediction results were compared to KEGG, GO, Swissprot, and Interpro databases. Functional annotation of protein-coding genes and transcripts was performed according to the best hit by BLASTp (v2.6.0+ , E-value ≤ 1e−5) searching of the SwissProt and TrEMBL (Bairoch and Apweiler 2000) protein databases. Motifs and domains were annotated by searching the Pfam, PRINTS, PROSITE, ProDom, and SMART InterPro (v. 29.0) databases using InterProScan (v. 5.16 55.0) (Jones et al. 2014). The Gene Ontology term for each gene was annotated by Blast2GO (Götz et al. 2008). Additionally, gene sets were mapped to KEGG (v. 53) (Kanehisa and Goto 2000) pathways to identify the best match classification for each gene (BLASTp E-value ≤ 1e−5).

### Reconstructed the ancestral chromosomes of Siluriformes

Genome and coding sequences of electric fishes and other related species were downloaded from the NCBI and ENSEMBL databases for a gene family analysis, including zebrafish (*D. rerio*, GCA_000002035.6), channel catfish [*I*. *punctatus*, GCA_001660625.2 (Liu et al. 2016b)], striped catfish [*Pangasianodon hypophthalmus*, GCA_009078355.1 (Kim et al. 2018)], Asian red-tailed catfish (*Hemibagrus wyckioides*, GCA_019097595.1), black bullhead catfish (*Ameiurus melas*, GCA_012411365.1), and Chinese large-mouth catfish [*Silurus meridionalis*, GCA_014805685.1 (Zheng et al. 2021)] (Supplementary Table S3). The predicted protein gene sets from *M. electricus* and six reference species were aligned using BLASTp (v2.6.0 +) with an E-value threshold of 1e−5, and the high-quality mapped genes were analyzed by OrthoMCL (v2.0.9) (Li et al. 2003) to define gene families. The proteins of single-copy orthologs gene families were aligned using MUSCLE (v3.8.31) (Edgar 2004), and four-fold degenerate synonymous sites (4D sites) were extracted from each alignment and concatenated to obtain a super gene for each species for phylogenetic tree construction using RAxML 8.2.4 (Stamatakis 2014). Masking was employed on *M. electricus* genome sequence to remove lineage-specific repetitive regions based on RepeatMasker and RepBase libraries by the RepeatMasker software suite. Then a whole-genome alignment between electric catfish assembly and the six species mentioned above was generated using LastZ (v1.1) (Harris 2007) with the parameter settings “*T* = *2 C* = *2 H* = *2000 Y* = *3400 L* = *6000 K* = *2200*”. After filtering the aligned blocks shorter than 2 Kb, the synteny between the two genomes was visualized by Circos (v0.69-6). Next, the pairwise alignment results were converted into the UCSC “chain” and “net” formats using the ChainNet algorithm (Kent et al. 2003). DESCHRAMBLER(Kim et al. 2017) was used for constructing chromosomes at 50 kb resolution. Then putative ancestors were inferred from orthologs maps by MGRA 2.2.1 (Avdeyev et al. 2016).

### Whole-genome duplication analysis

MCscanX (Wang et al. 2012) was used to detect syntenic blocks (regions with at least five collinear genes) between *M. electricus*, *E. electricus*, and *I. punctatus* based on the all-to-all BLASTp (v2.6.0+ , E-value ≤ 1e−10) results. Then the protein sequences of homologous gene pairs in the syntenic region were extracted and aligned using the MUSCLE (v3.8.31) program (Edgar 2004). Subsequently, the protein sequence alignments were converted into CDS files, and four-fold degenerate nucleotide sites that underwent transversions (4DTv distance) values of paralogous pairs within species and of orthologous pairs between species were calculated based on the CDS alignments, accompanying the correction of the HKY model (Hasegawa et al. 1985).

### Expansion and contraction of gene family analysis

Genome and coding sequences of electric fishes and other related species were downloaded from the NCBI and ENSEMBL databases for a gene family analysis, including *E. electricus*, *Paramormyrops kingsleyae*, *I. punctatus*, *P. hypophthalmus*, *D. rerio*, *Scleropages formosus*, and *L. oculatus*. The predicted protein gene sets from *M. electricus* and these seven reference species were aligned using BLASTp (v2.6.0+) with an E-value threshold of 1e−5, and the high-quality mapped genes were analyzed by OrthoMCL (v2.0.9) (Li et al. 2003) to define gene families. The proteins of single-copy orthologs gene families were aligned using MUSCLE (v3.8.31) (Edgar 2004), and four-fold degenerate synonymous sites (4D sites) were extracted from each alignment and concatenated to obtain a super gene for each species for phylogenetic tree construction using RAxML 8.2.4 (Stamatakis 2014). We obtained divergent times for all pairs of species in the phylogenetic tree using r8s version 1.71 (Sanderson 2003). The r8s results were calibrated with the teleost fossil records accessed from TimeTree website (http://www.timetree.org/, calibration divergence times used were for species *E. electricus* and *P. hypophthalmus*: 165–117 Mya). With the calibrated results as input, the MCMCtree (v4.5) in the PAML (v4.8) (Yang 2007) package was used to estimate species divergence time. Next, CAFE (v 4.2.1) (Han et al. 2013) was carried out with default parameters to define the expansion and contraction of gene families. Gene families exhibiting expansion and contraction were mapped to GO terms for an enrichment analysis.

### Detection of positively selected genes (PSGs)

To identify PSGs in *M*. *electricus* and the other three electric species, genes in COGs were extracted and then aligned using MUSCLE (v3.8.31) software (Edgar 2004). The Codeml package in the PAML (v4.8) (Yang 2007) was used to compute the dN/dS ratio of these alignments under branch site selection model, where the three electric species were designated as foreground branches. Only those genes were selected significantly positive which showed positive selection on the foreground branch, but negative or neutral selection on the background branch (using likelihood ratio test at a 5% significance level). These PSGs were further mapped to GO terms for an enrichment analysis.

### Genomic convergence analysis

To detect genome-wide sequence convergence between electric lineages, we followed both ∆SSLS (Parker et al. 2013) method and CCS method (Xu et al. 2017). In the ∆SSLS method, the CDS of every COG was built and aligned as codons using MUSCLE (v3.8.31) (Edgar 2004); then any ambiguously aligned sites and codons with excessive numbers of gaps were removed from each gene alignment using GBlocks v.0.91b (Talavera and Castresana 2007). Following the method of Zou and Zhang (Zou and Zhang 2015), the phylogenetic tree based on single-copy gene generation was used as a species tree (hereafter termed H0, Supplementary Fig. S1A). Then a first hypothetical topology was generated in which the electric species were forcibly clustered into one group; this topology was used as H1 (Supplementary Fig. S1A). A second hypothetical topology was generated in which the two groups of electric taxa were not clustered but otherwise exhibited the same amount of phylogenetic distortion from H0 as does H1; this topology was used as H1′ (Supplementary Fig. S1A). To assess the alignment data relative to the H0, H1, and H1, we fitted the data using the Codeml program in the PAML (v4.8) (Yang 2007) package under the WAG + γ model with estimated amino acid frequencies to generate the site-wise log-likelihood support (SSLS). Then the comparison log-likelihood differences per site (∆L) was calculated, with significantly negative ∆L_H0−H1_ values (or ∆L_H0−H1'_ values) indicating that the evolution of the protein favors H1 (or H1′) over H0 (Parker et al. 2013). The protein evolutionary tendency was considered significant (Kolmogorov–Smirnov test, *P* value < 0.05) only when the frequency distribution of ∆L_H0-H1_ did not overlap with that of ∆L_H0−H1′_ (Zou and Zhang 2015).

For the CCS method, ancestral protein sequences were first reconstructed for single-copy orthologs detected among eight species using the Codeml program in the PAML (v4.8) (Yang 2007) package. Observed convergent amino acid sites among *M. electricus*, *E. electricus*, and *P. kingsleyae* with rules as follow (Hu et al. 2017): (i) the amino acid residues of both the extant target lineages were identical; (ii) amino acid change was inferred to have occurred between the extant target lineages and the most recent common ancestor (MRCA) of each two of them.

Concurrently, the expected number of these two substitutions between electric fish species and other species’ predicted MRCA was calculated under the JTT-f_genes_ amino acid substitution model (Jones et al. 1992). To filter out noise from chance amino acid substitutions (Thomas and Hahn 2015; Zou and Zhang 2015), Poisson's tests were performed on observations and estimates to verify significant differences (*P* value < 0.05). Finally, genes within both gene sets of non-random convergent (or parallel) genes and PSGs were inferred to have undergone adaptive convergence.

### Opsin and taste receptor genes identification

With reference to Ding et al. (Ding et al. 2021), zebrafish opsin protein genes (*opsin*) were downloaded from ENSEMBL database (http://www.ensembl.org), including *rhodopsin* (*rh1*): ENSDARP00000011562; green-sensitive (*rh2*), ENSDARP00000001158, ENSDARP00000011837, ENSDARP00000001943, ENSDARP000000009794; short wavelength-sensitive 1 (*sws1*): ENSDARP00000067159; short wavelength-sensitive 2 (*sws2*): ENSDARP00000144766; long wavelength-sensitive (*lws*), including *lws-1*: ENSDARP00000065940, *lws-2*: ENSDARP00000149112. Three types of taste receptor genes (*tr*), including sour, sweet-umami and bitter taste receptor genes were downloaded from the Uniprot database (https://www.uniprot.org) (Supplementary Table S5).

Homology predictions of the gene sets above in the electric catfish and the closely related and zebrafish *D. rerio*, electric eel *E. electricus*, black bullhead catfish *A. melas*, Asian red-tailed catfish *H. wyckioides*, channel catfish *I. punctatus* (Liu et al. 2016b), striped catfish *P. hypophthalmus* (Kim et al. 2018), Chinese large-mouth catfish *S. meridionalis* (Zheng et al. 2021), elephantfish *P. kingsleyae*, Asian bonytongue *S. formosus*, and spotted gar *L. oculatus* were performed using BLAT software (version 0.36) (Kent 2002) against the reference protein sequences. The best alignments were extended 2Kb on the both sides, then the possible gene structures were predicted using GeneWise software (version 2.4.1) (Birney et al. 2004). Then predicted protein sequences were obtained and validated by comparing the protein sequences to the non-redundant (NR) [http://ftp.ncbi.nih.gov/blast/db/FASTA (accessed on 19 Feb 2016)] based on BLASTp E-values ≤ 1e−05.

Protein sequences were then aligned by the MAFFT (v7.237) (Katoh and Standley 2013) with auto module. Maximum likelihood (ML) trees were constructed using the RAxML 8.2.4 (Stamatakis 2014) with 1000 bootstraps. For taste receptor genes, identified *pkd2l1* genes were used as outgroups. Subsequently the trees were visualized using FigTree (http://tree.bio.ed.ac.uk/software/figtree) and the Interactive Tree Of Life online website (iTOL, https://itol.embl.de) (Letunic and Bork 2021).

### Estimates of effective population size

The demographic history of *M*. *electricus* was inferred PSMC method (Li and Durbin 2011). The quality and barcode-trimmed stLFR data was mapped to the assembly using BWA v. 0.7.12 (Li and Durbin 2009) with default parameters, then the SNP variants were called using bcftools-1.4 (Danecek et al. 2021) (mpileup parameters: *–Ou –I –C 30 –d 100*; call parameters: *–c –Ov*). The vcfutils.pl program of bcftools-1.4 (Danecek et al. 2021) was further applied to generate the diploid consensus sequences. Then the input file for PSMC modeling was generated with the program ‘fq2psmcfa’ (*–q 20*); then the population size history was inferred with the program ‘psmc’ (*–N25 –t15 –r5 –p 4* + 25*2 + *4* + *6*) in PSMC v. 0.6.5-r67 (Li and Durbin 2011). Bootstrapping was conducted by randomly sampling with replacement 5-Mb sequence segments during 100 bootstrap replicates. The generation time (*g*) was assumed to be two year, according to the equation “*g* = *a* + [*s*/(1 − *s*)]” (Liu et al. 2016b), where *s* is the expected adult survival rate which is roughly recorded as 0.5 (Goli Bi et al. 2019), and *a* is the sexual maturation age that is one year (https://www.fishbase.se/summary/Malapterurus-electricus). The reconstructed population history was then plotted, and the mutation rate per site per year was set at 3 × 10^–9^ estimated by r8s version 1.71 (Sanderson 2003), making the mutation rate per site per generation (*g* = 2) 6 × 10^–9^ (the *μ* value). We then used Sequential Markov Coalescent + Plenty of Unlabeled Samples (SMC++, v1.15.5) (Terhorst et al. 2017) to estimate fitted models to infer a more recent demographic history for *M*. *electricus*. The chromosome-wise vcf file generated in the previous step was continually converted to SMC input files using the vcf2smc module. Then SMC++ *estimate* model was run with a smooth spline model, 10 knots in internal and cross-validated 5 times (–folds 5, –spline cubic, –konts 10) with the same mutation rate (Terhorst et al. 2017). We finally plotted the data using the plot command with a generation time of two years.

## Results

### Genome assembly and annotation

**Fig. 1:**
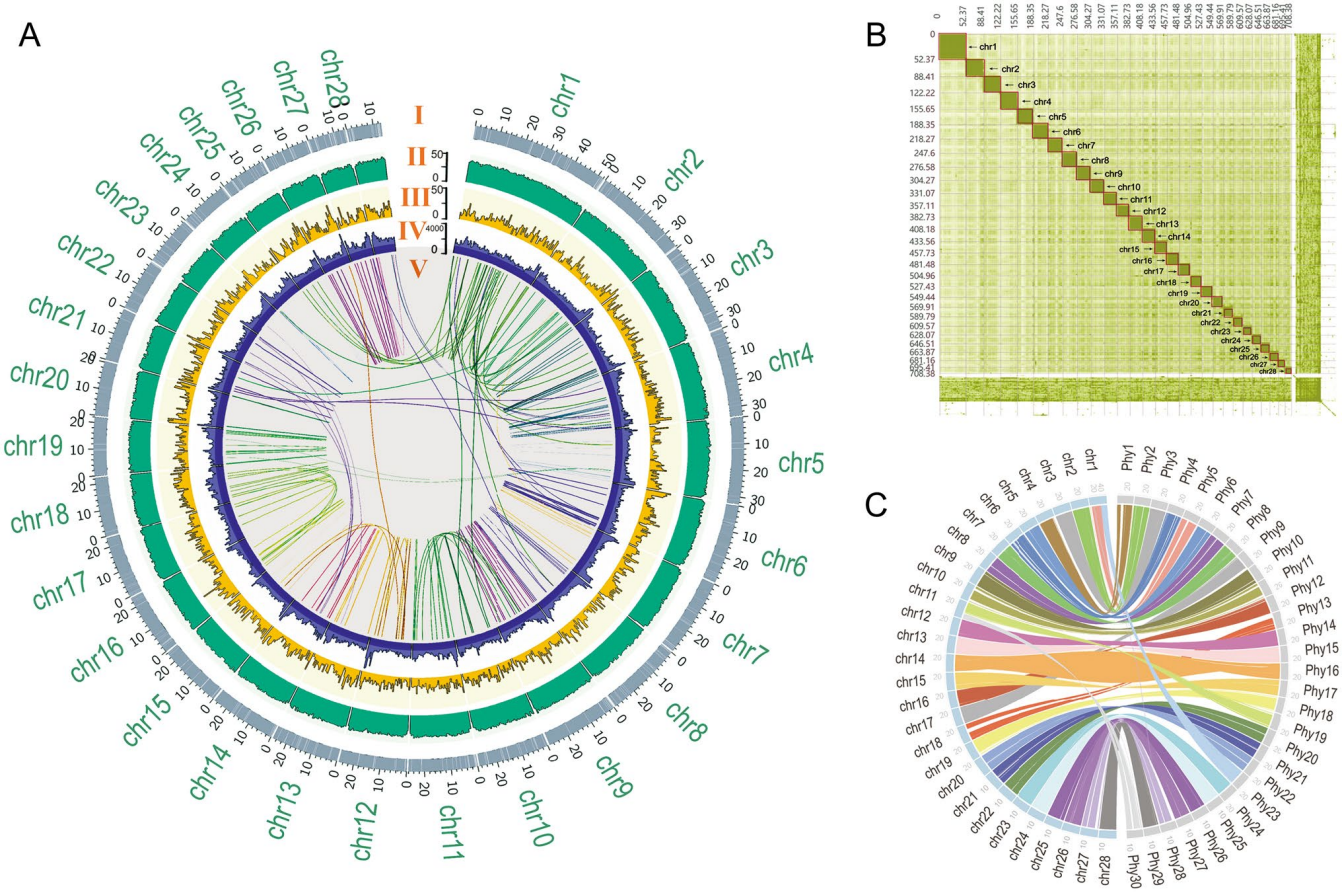
Genomic landscape of *M*. *electricus* chromosomes. **A**, The representation of the chromosome scaffold assembly information of *M. electricus*. From outside to the inside ring: (I) Physical map of 28 assembled chromosomes (Mb scale) numbered according to size. The scaffolds and gaps on each chromosome are shown in gray and white segments, respectively. (II) GC content represented by percentage of G + C bases in 500 kb windows. (III) Gene density represented by number of genes in 500 kb windows. (IV) Repeat density represented by proportion of genomic regions covered by repetitive sequences in 500 kb windows. (V) Syntenic blocks are depicted by connected lines. **B**, Heatmap of interactions within and among chromosomes according to Hi-C analysis. Chromosomes size scaffolds are indicated by the red frames and numbered according to size. **C**, The collinear represents the collinear relationships between *M*. *electricus* and *P*. *hypophthalmus*

We used 118.87 Gb (~ 82 ×) stLFR data of *M. electricus*, generated on the BGISEQ-500 platform (Supplementary Table S1), to obtain a preliminary genome assembly of 796.45 Mb with a scaffold N50 of 3.58 Mb (Table 1; Supplementary Table S2, S6). To further obtain the chromosome-level assembly, 311.10 Gb Hi-C data were used to anchor the initial assembly onto 28 chromosomes with a 796.75 Mb genome size (Fig. 1; Table 1; Supplementary Table S1, S7, S8). The heterozygosity rate of this genome was 0.043%, consistent with the k-mer estimate analysis (0.05%) (Supplementary Figs. S2, S3; Table S9, S10), and the assembly error ratio was 0.001%, with 98.68% of sequencing reads mapping ratio indicating a high-quality and intact assembly.

**Table 1:** Assembly and annotation of the *M. electricus* genome

Field	Parameter	Contents
Genome sequencing	stLFR reads	118.87 Gb (~ 82 ×)
	Hi-C reads	307.07 Gb (~ 211 ×)
	Estimation of genome size	830.5 Mb
Genome assembly	Total length of genome assembly	796.75 Mb
	GC content	40.45%
	BUSCO	87.2%
	Number of chromosomes	28
	Average chromosomes size	25.31 Mb
Repeat and gene annotation	Repetitive sequences	264.1 Mb (33.15%)
	Number of predicted genes	19,985
	Overall functional annotation	19,011 (95.13%)

We identified nearly 264.1 Mb of repetitive sequences, representing 33.15% of the assembly (Table 1; Supplementary Fig. S4; Table S11, S12). DNA transposons were the most abundant, accounting for 19.52%, followed by long terminal repeat (LTR) retrotransposons (11.76%) (Table 1; Supplementary Table S11, S12). A total of 19,985 protein-coding genes were annotated, with an average gene length of 16,149.9 bp and an average of 9.06 exons (Supplementary Table S13), similar to published genomes (e.g., *E. electricus* and *P. hypophthalmus;* see Supplementary Table S14). The results show that 95.13% (19,011/19,985) of these genes had recorded functional descriptions in databases, such as SwissProt, KEGG, TrEMBL, and InterPro, accounting for 90.57%, 85.98%, 94.76%, and 91.49% of the total gene set, respectively (Supplementary Table S15).

### Reconstruction of the ancestral Siluriformes karyotype

**Fig. 2:**
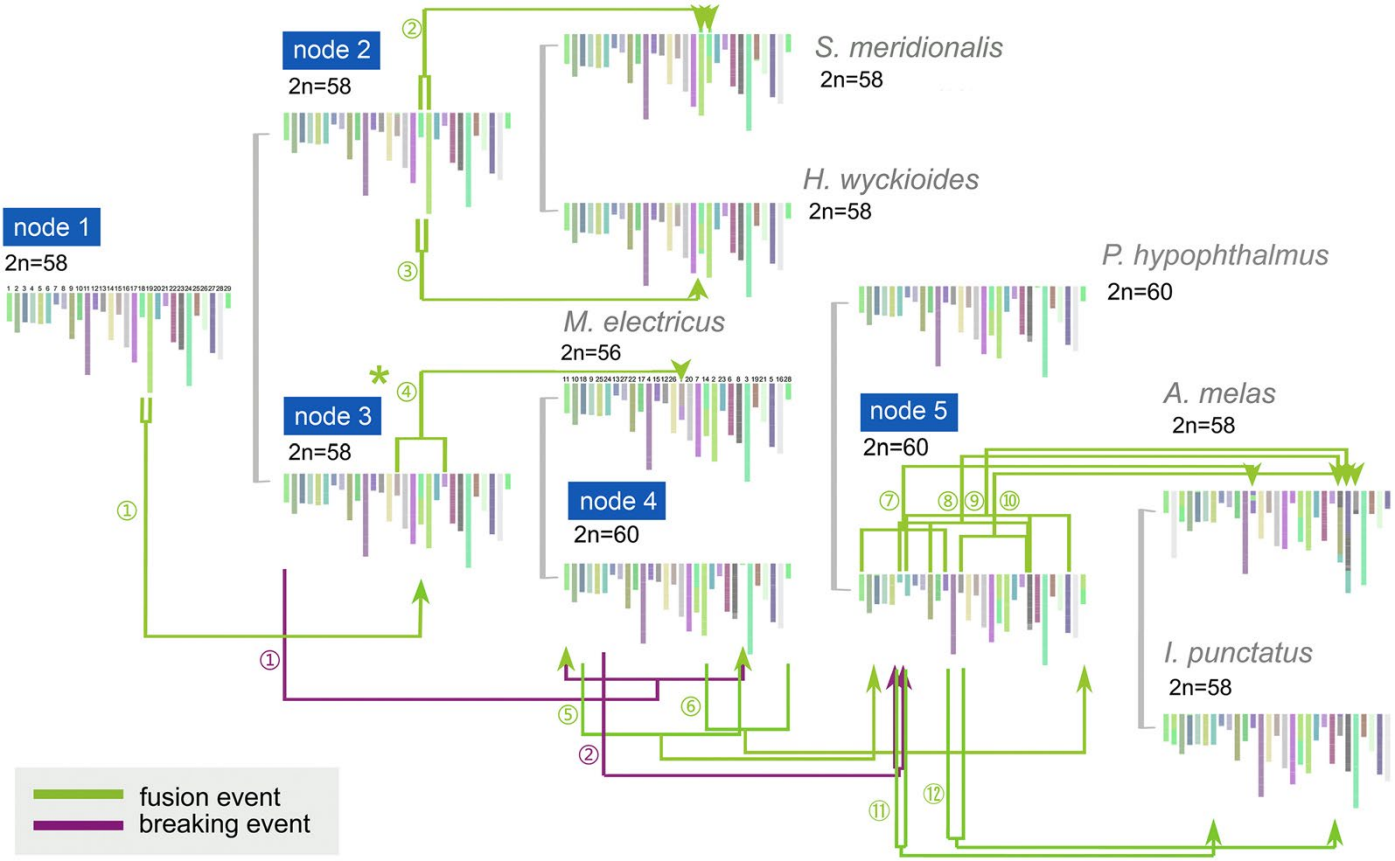
Reconstruction of karyotype evolution in catfishes (order Siluriformes), including the karyotypes of six Siluriformes taxa (*M. electricus*, *I. punctatus*, *P*. *hypophthalmus*, *H. wyckioides*, *A. melas*, and *S. meridionalis*) as well as reconstructed ancestral karyotypes based on 267 shared homologous synteny blocks (HSBs). Each synteny block is represented using a line segment that is color-coded based on its position in the ancestral genome of all species. Chromosome fusion and breaking events are indicated by green and purple connecting lines, respectively. The green asterisk labels a major fusion event that occurred in *M. electricus*

Using six chromosome-level Siluriformes fish genomes, including *M. electricus*, the channel catfish (*I. punctatus*), the striped catfish (*P. hypophthalmus*), the Asian red-tailed catfish (*H. wyckioides*), the black bullhead catfish (*A. melas*), the Chinese large-mouth catfish (*S. meridionalis*), and zebrafish (*D. rerio*) as the outgroup, we reconstructed the ancestral Siluriformes karyotype (Fig. 2). A total of 267 common shared homologous synteny blocks (HSBs) were identified at 50-kb resolution, covering 34.22%, 31.68%, 30.42%, 30.68%, 31.63%, and 32.85% of the *M. electricus*, *P. hypophthalmus*, *H. wyckioides*, *A. melas*, *S. meridionalis*, and *I. punctatus* genomes, respectively (Supplementary Table S16). Using these HSBs, 29 ancestral chromosomes were reconstructed for the last common ancestor (LCA) of all Siluriformes, while the LCA of *P. hypophthalmus*, *A. melas*, and *I. punctatus* has 30 ancestral chromosomes. Synteny analysis revealed that the assigned chromosomes of *M. electricus* (2n = 56) and *P. hypophthalmus* (2n = 60) are highly homologous (Fig. 1C), except for a fusion of the ancestral chromosomes in chromosome 1 of *M. electricus* and a fission of chromosome in the ancestor of *P. hypophthalmus*, *A. melas*, and *I. punctatus*.

### Phylogenetic relationship and estimation of divergence time

To reveal the evolutionary history of electric fishes, we selected eight fish species to explore the origin–time of different electric fish species, including two strongly electric fishes (*M. electricus* and *E. electricus*), one weakly electric bony fish elephantnose fish (*P. kingsleyae*) and five non-electric fishes: striped catfish (*P. hypophthalmus*), channel catfish (*I. punctatus*), zebrafish (*D. rerio*), Asian bony tongue (*S. formosus*), and as an outgroup, the spotted gar (*L. oculatus*).

**Fig. 3:**
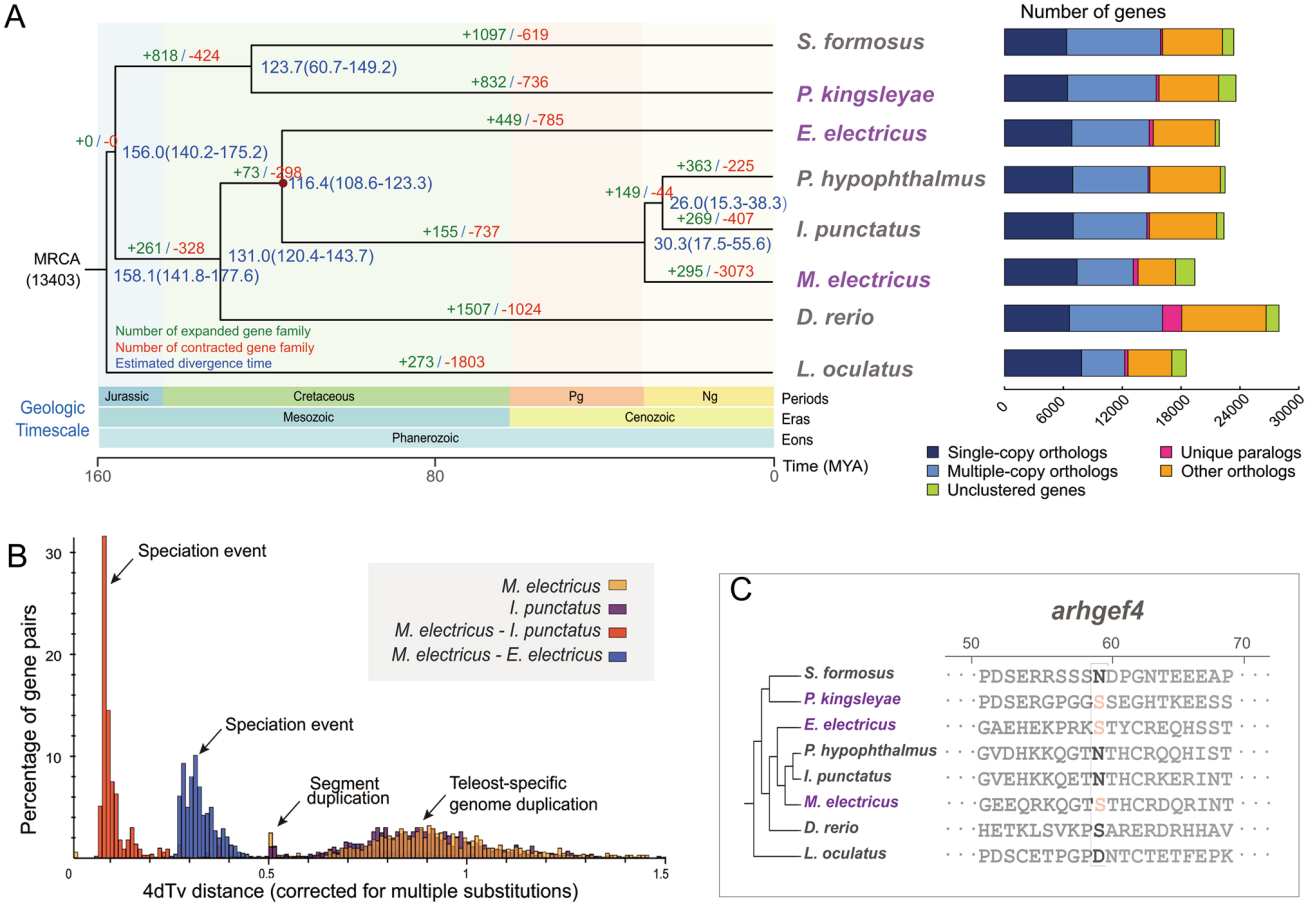
Species divergence and evolution analysis of *M. electricus*. **A**, Gene family analysis. On the left is a phylogenetic tree constructed with single copy genes, with purple and gray species names indicating electric and non-electric classification, respectively. Numbers at the node positions represent the divergence time of each species in millions of years ago (Mya). The numbers in parentheses indicate the confidence interval of the divergence time, which can be used to estimate the divergence time of target species and other species. The red dots are the calibration time used to correct the time of species divergence, which were obtained from the Timetree website (http://www.timetree.org/). Green and red numbers represent the number of gene families that expanded and contracted during evolution, respectively. On the left is the summary of gene family analysis. Statistics of single-copy orthologs, multiple-copy orthologs, unique paralogs, other orthologs and unclustered gene numbers in *E. electricus*, *P. kingsleyae*, *I. punctatus*, *P. hypophthalmus*, *D. rerio*, and *S. formosus*, using *L. oculatus* as an outgroup. **B**, Distribution of the transversions on four-fold degenerate synonymous sites (4DTv) distances among paralogs for *M. electricus*, *E. electricus*, and *I. punctatus*. **C**, Sequence alignments of *arhgef4* among electric and non-electric fish species. The box identifies the loci with convergent signals in the electric fish lineages

The phylogenetic relationship reconstructed from 4938 single-copy gene families of these species revealed that the electric *M. electricus* shared an ancestor with the non-electric *I. punctatus* and *P. hypophthalmus* ~ 30 million years ago (Mya) (Fig. 3A). We also investigated the 4DTv (the transversion rate at four-fold degenerate third-codon positions) distribution for gene pairs in syntenic blocks between *M. electricus*, *I. punctatus*, and *E. electricus*. Briefly, the peaks at approximately 0.90 support the teleost-specific genome duplication (TGD) event 320 Mya (Ravi and Venkatesh 2018) (Fig. 3B), while the peaks at ~ 0.51 indicate possible segment duplications in *M. electricus* and *I. punctatus*. Moreover, a speciation peak between *M. electricus* and *I. punctatus* at ~ 0.08 occurred after the speciation peak between *M. electricus* and *E. electricus* (~ 0.31) (Fig. 3B).

### Gene family analysis on biological discharge of electric fishes

Using the gene sets of *M. electricus* and other two electric fish *E. electricus* and *P. kingsleyae*, and five related non-electric fish *I. punctatus*, *P. hypophthalmus*, *D. rerio*, *S. formosus* and *L. oculatus*, we identified a total of 13,016 gene families, among which 139 were specific to *M. electricus*, containing 348 genes (Supplementary Table S17). These 348 *M. electricus*-specific genes may be closely associated with the unique traits and characteristics of this species, suggesting potential genetic adaptations and evolutionary processes that contribute to the distinct features of *M. electricus*. We found functional enrichments in these specific gene families, including ATPase inhibitor activity (GO:0042030), alpha-1,6-mannosylglycoprotein 6-beta-N-acetylglucosaminyltransferase activity (GO:0030144), syntaxin binding (GO:0019905), voltage-gated anion channel activity (GO:0008308), and DNA-dependent ATPase activity (GO:0008094) (Supplementary Table S18). Additionally, we identified 295 significantly expanded gene families in *M. electricus* (*P* value < 0.05), enriched for ionotropic glutamate receptor activity, extracellular glutamate-gated ion channel activity, magnesium ion binding, and transferase activity (Supplementary Table S19).

To further explore the genetic mechanism of bioelectrogenesis in electric fish, we identified 256 positively selected genes (PSGs) in three electric fishes (reported by Moreno-Hagelsieb and Latimer 2007), using five non-electric fishes as the background (Supplementary Table S20). Among these 256 PSGs, 12 PSGs contained the parallel amino acid (AA) mutation sites in the three electric fish species (*M. electricus*, *E. electricus*, and *P. kingsleyae*) detected by the conserved convergent signal (CCS) method (Xu et al. 2017). The encoding gene for a rho guanine nucleotide exchange factor 4-like protein, *arhgef4* was expressed in electric organs (EOs), with the expression of 1.41 fragments per kilobase of transcript per million fragments mapped (FPKM) (Fig. 3C).

### The visual and chemosensory gene repertoire of catfishes

**Fig. 4:**
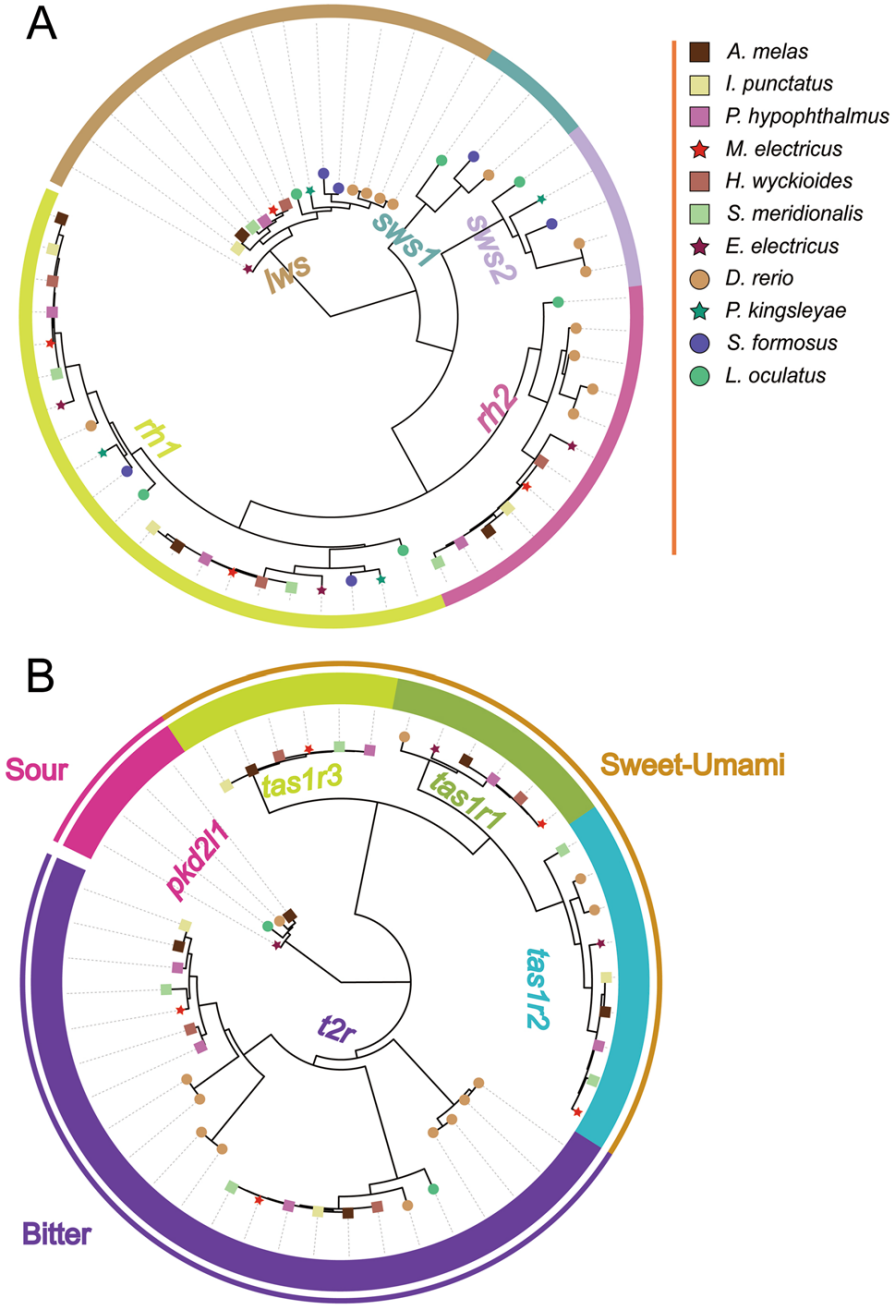
Maximum likelihood (ML) tree of two gene families related to the electric feature. **A**, ML tree of the *opsin* gene identified form 11 fishes. **B**, ML tree of the taste receptor (TR) gene identified from 11 fishes, with *pkd2l1* sequences identified used as outgroups. The 11 species are indicated by different node decorations

To investigate the evolution of vision- and taste-related gene families in electric catfish, we collected ten *opsin* sequences and 24 taste receptor genes to identify orthologous genes in electric catfish and other closely related species (Supplementary Table S3). A total of 56 *opsin* sequences were annotated, including 21 *rh1*, 12 *rh2*, three *sws1*, five *sws2*, and 15 *lws* (Fig. 4A). Phylogenetic analysis revealed two major branches of *opsin* genes: rod opsin (*rh1*) and cone opsin (*rh2*, *lws*, *sws1*, and *sws2*). One single-exon *rh1* and one rh1 with five exons (*exrho*) were found in all catfishes (*M. electricus*, *I. punctatus*, *P. hypophthalmus*, *H. wyckioides*, *A. melas*, and *S. meridionalis*), electric eels, bony tongues (*P. kingsleyae* and *S. formosus*), and spotted gar (*L. oculatus*), and clustered into two branches (Fig. 4A). Only one *lws* gene was found in catfishes and electric eel, neither of which had *sws1* or *sws2*. In addition to vision genes, we identified 48 taste receptors in the genome of electric catfish, including four *pkd2l1*, six *t1r1*, nine *t1r2*, six *t1r3*, and 23 *t2r* (Fig. 4B). Phylogenetic analysis showed that the genes clustered into two large branches, *t1r* and *t2r*, with the *t1r* branch first dividing into *t1r3* followed by the *t1r1* and *t1r2* branches. All three *t1r* genes were found in all catfish, except for the large-mouth catfish *S. meridionalis* and the channel catfish, which lack *t1r1*, and the Asian red-tailed catfish *H. wyckioides*, which lacks *t1r2*; *t1r3* and *t2r*, were not found in the electric eel genome.

### Population history of *M. electricus*

**Fig. 5:**
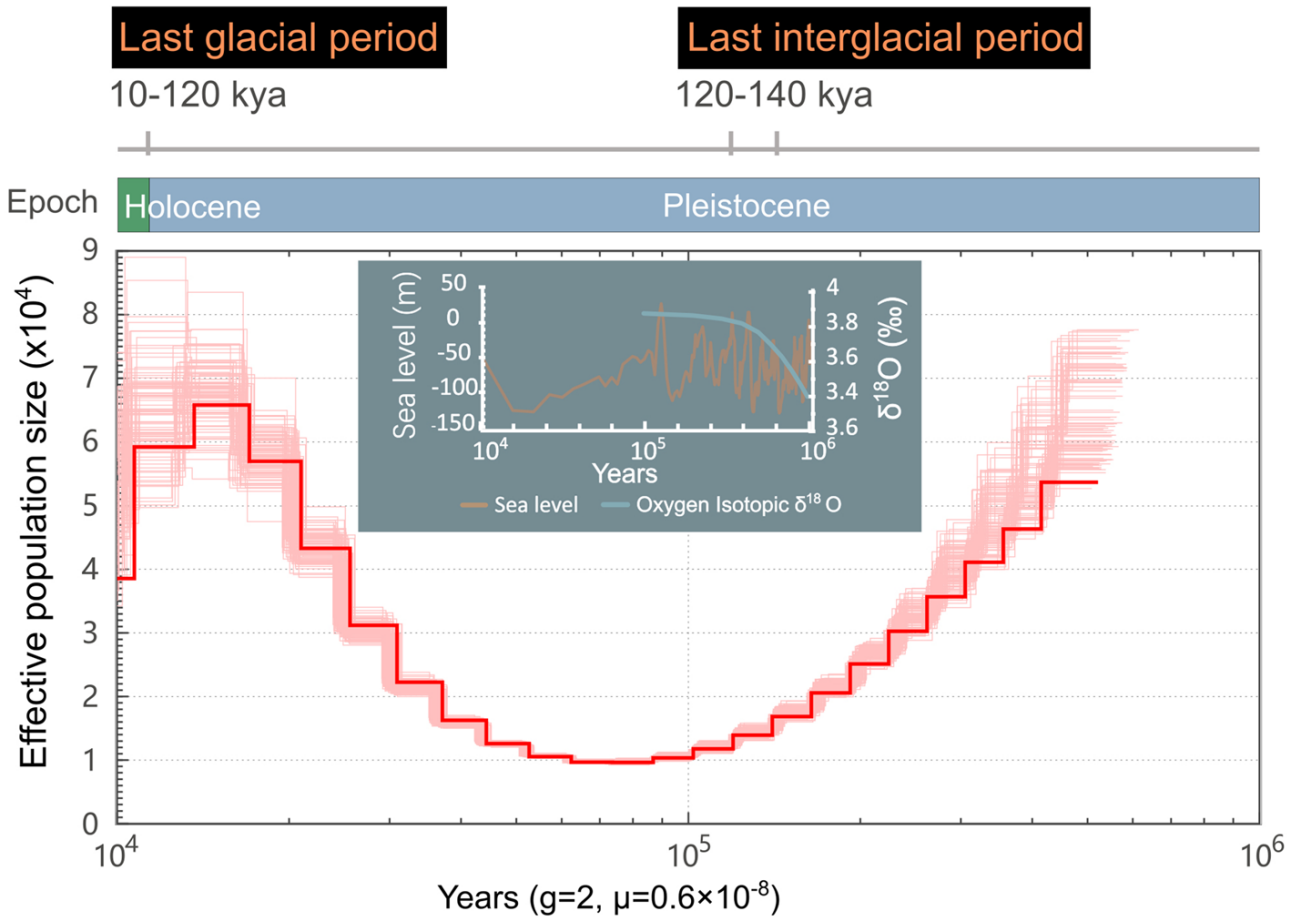
Inferred historical effective population size of *M. electricus* revealed by PSMC analysis. The inferred population size is shown as a bold red line, surrounded by pink lines that shows the population size estimate generated after 100 rounds of bootstrapping. The bar above shows the last two geological periods: the early Holocene (~ 8000–11,650 years ago) and the late Pleistocene (~ 12,000–2,480,000 years ago); line segments show the last glacial period (~ 11,700–120,000 years ago) and the last interglacial period (~ 116,000–129,000 years ago). The global climate folding line graph (sea level and oxygen isotope δ^18^ content) is from Miller et al. (2005)

We inferred the demographic history of *M. electricus* by the pairwise sequentially Markovian coalescent (PSMC) (Li and Durbin 2011) as well as the SMC++ (Terhorst et al. 2017) (Fig. 5; Supplementary Fig. S5). Our data revealed distinct demographic trends from ~ 500,000 to 10,000 years ago, in which a bottleneck was shown ∼70,000–100,000 years ago with a minimum of ∼10,000 individuals, followed by an immediate expansion of population size that peaked between 10,000 and 20,000 years ago with ~ 70,000 individuals (Fig. 5). The SMC++ result also suggests a bottleneck ~ 70,000–100,000 years ago and a recent (< 50,000 years ago) population expansion (Supplementary Fig. S5).

## Discussion

Here, we have reported a chromosome-level genome of *M. electricus*, resulting in a complete genome assembly and comprehensive annotation. The genome size and the GC content of the *M. electricus* genome were similar to those of catfish species and had good collinearity with the neighboring species *P. hypophthalmus*. The number of coding genes was similar to other Siluriformes species. This newly assembled chromosome-level genome assembly will not only help to understand the genetic basis of electric discharge but also facilitate the exploration of the mechanisms underlying other physiological processes and the evolution of electric fish. The current assembly result of our study provides a solid foundation for further genetic and evolutionary research on *M. electricus*. However, given the complexity of the *M. electricus* genome, the current assembly still requires further improvement using updated sequencing technologies, such as long-read sequencing generated by third-generation sequencing technologies. These advanced sequencing techniques and assembly strategies will provide valuable insights into the structure and function of the *M. electricus* genome, furthering our understanding of its biology and evolution.

### Variations in karyotypes in Siluriformes species

The teleost ancestor had 24 or 25 chromosomes (Nakatani and McLysaght 2017; Naruse et al. 2004), followed by a series of genome-wide replications, chromosome fissions/fusions, deletions, and rearrangements (Kasahara et al. 2007; Parey et al. 2022; Ravi and Venkatesh 2018; Woods et al. 2005; Zhu et al. 2022). In Siluriformes, an ancestral karyotype is 2n = 58 (LeGrande 1981) and varied roughly between 2n = 24 and 2n = 60 (see Supplementary Table S21). Since karyotypes of *S. meridionalis* and *H. wyckioides* and their LCA with *M. electricus* were all 2n = 58, we suggest that a major fusion event caused *M. electricus* to exhibit a 2n = 56 karyotype. This suggests that there were changes in the number of chromosomes present in the genome of Siluriformes over time.

### Gene *arhgef4* relates to convergent evolution of electric organ

The expanded gene families in *M. electricus*, whose functions are enriched on glutamate receptor activity, emphasize their close correlation with their biological discharge functions. Previous studies have shown significant enrichment of overexpressed genes involved in ionotropic glutamate receptor activity and extracellular glutamate-gated ion channel activity pathways in the brain and spinal cord of electric eels (Traeger et al. 2015), as well as differentially expressed genes in the electrical organ (EO) of three African weakly electric fish when compared to the skeletal muscle (SM) dataset (Lamanna et al. 2015).

Previous studies have shown that tree-based ΔSSLS method produce false positives in detecting convergence on whole-genome data (Thomas and Hahn 2015; Xu et al. 2020; Zou and Zhang 2015). In many cases, critical but small-scale amino acid convergent changes within protein sequences may not be strongly represented in the overall gene tree. Furthermore, this method cannot clarify whether the convergence of these proteins occurred independently as a result of the electric organ discharge function in different species. In our study, we used the CCS method (Xu et al. 2017) for identifying convergent evolution at the amino acid level. With this approach, we identified a positively selected gene, *arhgef4*, with one convergent amino acid site. The *arhgef* gene family, involved in signal transduction through the GPCR signaling pathway, has been shown to be up-regulated in EOs compared to SMs (Gallant and O’Connell 2020; Gallant et al. 2014; Lamanna et al. 2015). A key step in biological electrogenesis is the degradation of the excitatory–contraction pathway, which prevents organs from twitching during discharge (Gallant et al. 2014; Zakon et al. 2006). In hepatic stellate cells, the human homolog of *arhgef4* mediates the reorganization of the actin cytoskeleton and plays a role in the expression of morphological genes (Zhang et al. 2019). Hippocampal neuronal cultures from *Arhgef4* (the mice homolog of *arhgef4*) KO mice show that the protein acts as a negative regulator in excitatory synapses (Yoo et al. 2020). Therefore, *arhgef* genes with convergent signaling may play a role in the biological electrogenesis and self-protection of electric fish organs.

### Visual and taste depictions of environmental adaptation in Siluriformes

The *sws1* and *sws2* genes are primarily responsible for short-wave sensitive vision, and short-wave light penetrates weakly in turbid water (Enright et al. 2015; Lin et al. 2017). As we report, the two short-wavelength-sensitive cone opsin genes were presumed lost in nocturnally active gymnotiforms and catfish (Liu et al. 2016a), possibly indicating that their shortwave vision is degraded by the ecological niche of turbid water (Fagbenro et al. 2001). All of the catfish species we examined had at least two *t1rs*, suggesting the taxon may have a highly developed sense of taste, which is consistent with behavioral findings (Bauer 1968). In contrast, the loss of some taste genes may be due to narrow feeding habits or species-specific feeding patterns that render taste unimportant (Feng et al. 2014; Zhao et al. 2010, 2012). As in catfish with other transduction pathways for bitter taste that do not depend on *t2r*, the taste response to L-arginine (a bitter substance) is mediated by a non-selective cation channel (Bigiani et al. 2003; Kumazawa et al. 1998; Shi and Zhang 2006), which may lead to reduced or absent copies of *t2r*.

### Global geological events affect historical effective population size of *M. electricus*

Effective population size (*N*_*e*_) is a central concept in evolutionary theory and is essential for understanding changes in gene frequencies in finite populations (Lee et al. 2020). It is possible to reconstruct the population size history of species based on the genome sequences of representatives of present-day species (Mather et al. 2020). Global glacial oscillations during the Pleistocene over the past 1.1 million years may have led to oscillatory changes in species populations (Aguilar et al. 2019). Dramatic geological changes over different geological periods, such as sea level fluctuations, can connect and disconnect water systems, promote habitat integration or isolation, and spawn new habitats (Arias et al. 2021), thereby altering species distributions. For example, the effective population of the short-nosed electric eel *Brachyhypopomus occidentalis* (Teleostei, Gymnotiformes) expanded rapidly between the final closure of the Isthmus of Panama between 2.8 and 3.5 Myr and declined during the Pleistocene ice-interglacial cyclone (Arias et al. 2021). The dynamic change of catfish population size was also consistent with that of other catfish species, such as the freshwater Wels catfish (*S. Glanis*) (Ozerov et al. 2020). The period of effective population sizes declined in the electric catfish (~ 70,000–100,000 years ago) is very close to earth's history of the last interglacial period (~ 116,000–129,000 years ago) (Nascimento et al. 2022) and the great population expansion might be associated with the end of quaternary glaciation at 10,000–20,000 years ago (Clark et al. 2009; Zachos et al. 2001). As a benthic tropical freshwater fish (Fagbenro et al. 2001), *M. electricus* populations were at low levels after the last interglacial (Eemian interglacial, 116,000–129,000 years ago), probably a period of increased global temperatures, higher connectivity of water bodies (Krijgsman et al. 2019; Ozerov et al. 2020), and greater interaction between freshwater and seawater, which affected freshwater-dwelling electric catfish.

### Supplementary Information

Below is the link to the electronic supplementary material.

Supplementary file1 (PDF 1096 KB)

Supplementary file2 (XLSX 41 KB)

## Data Availability

The genome assembly of *M*. *electricus* has been deposited in the CNGB Nucleotide Sequence Archive (https://db.cngb.org/cnsa/) under the Project ID CNP0004026.
