# Supplementary material for: A chromosome-level genome of electric catfish (Malapterurus electricus) provided new insights into order Siluriformes evolution
Source: Mar Life Sci Technol. 2023 Dec 14;6(1):1–14. doi: 10.1007/s42995-023-00197-8 (PMC10901758; doi:10.1007/s42995-023-00197-8)
Supplement: Supplementary file 1 — Supplementary file1 (PDF 1096 KB) [file 42995_2023_197_MOESM1_ESM.pdf]

Supplementary Figures

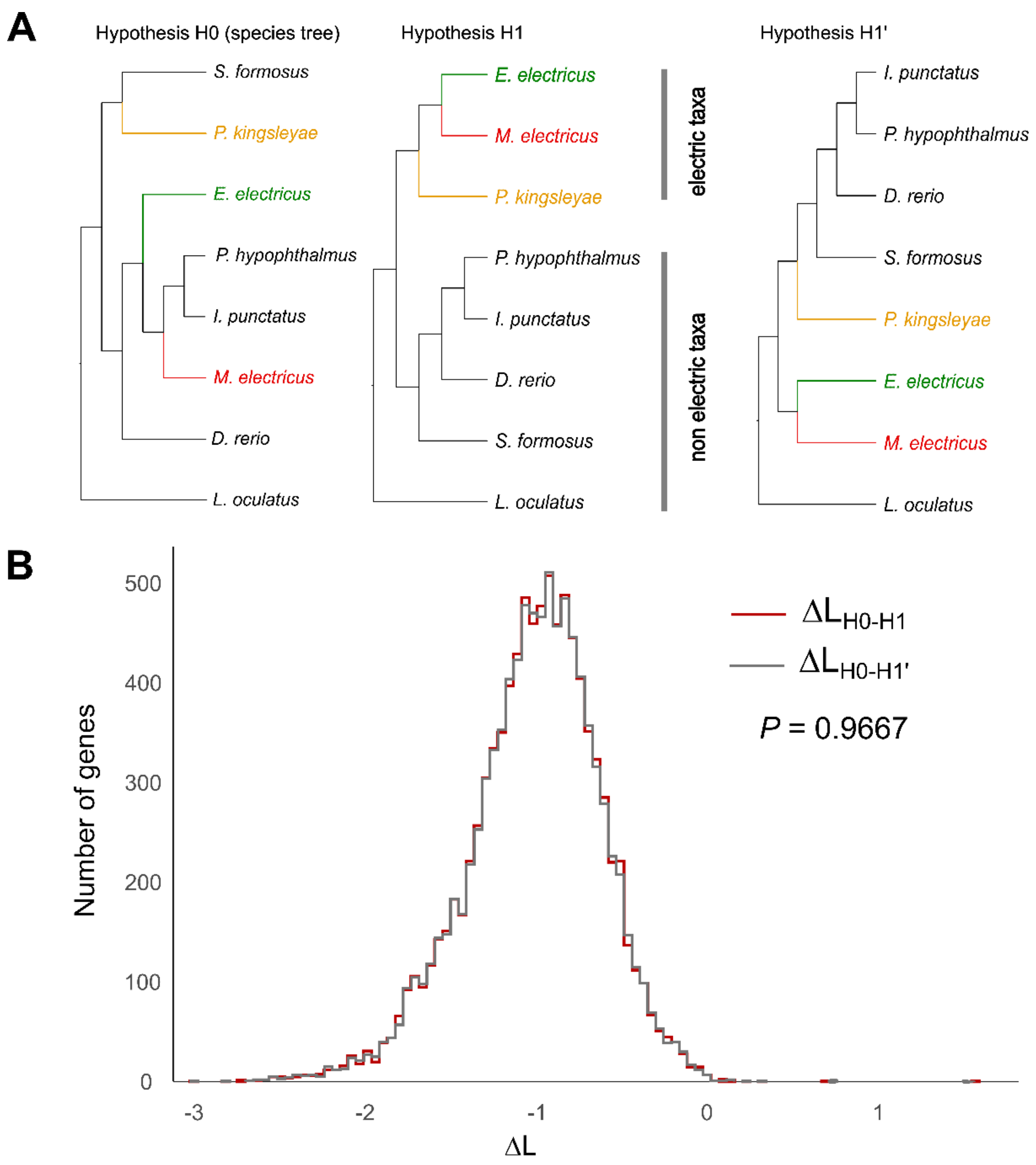

Fig. S1. Convergence hypotheses and genomic distribution of support by ΔSSLS method. A, Hypotheses and corresponding tree topologies. H0, species tree; H1, clustering of the electric taxa; H1', electric taxa not clustered but otherwise exhibited the same amount of phylogenetic distortion from H0 as does H1. Electric species are indicated with different colors. B, Frequency distributions of $\Delta L_{H0\text{-}H1}$ and $\Delta L_{H0\text{-}H1'}$ among the proteins are not significantly different. ΔL refers to the per site logarithm of the likelihood ratio between two hypotheses for a protein. The P-values is from Kolmogorov-Smirnov tests.

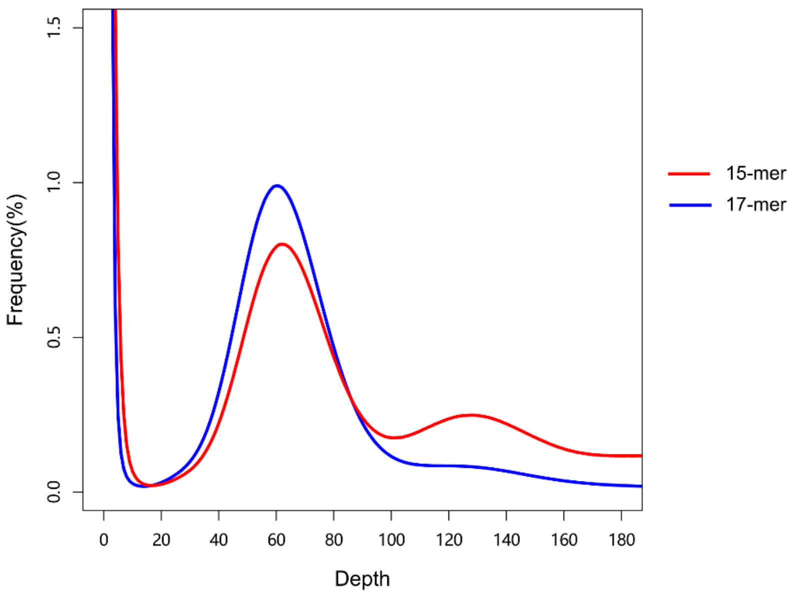

Fig. S2. Estimated genome size of *M. electricus* from k-mer analysis. The figure shows the 15-mer and 17-mer frequency distribution of *M. electricus*. The genome size was estimated from the depth of the main peak, genome size = k-mer_num/peak_depth. Here the k-mer number is 51,533,475,112, when the k-mer is 15, the peak depth is 62, and the estimated genome size is therefore 831,185,082 bp.

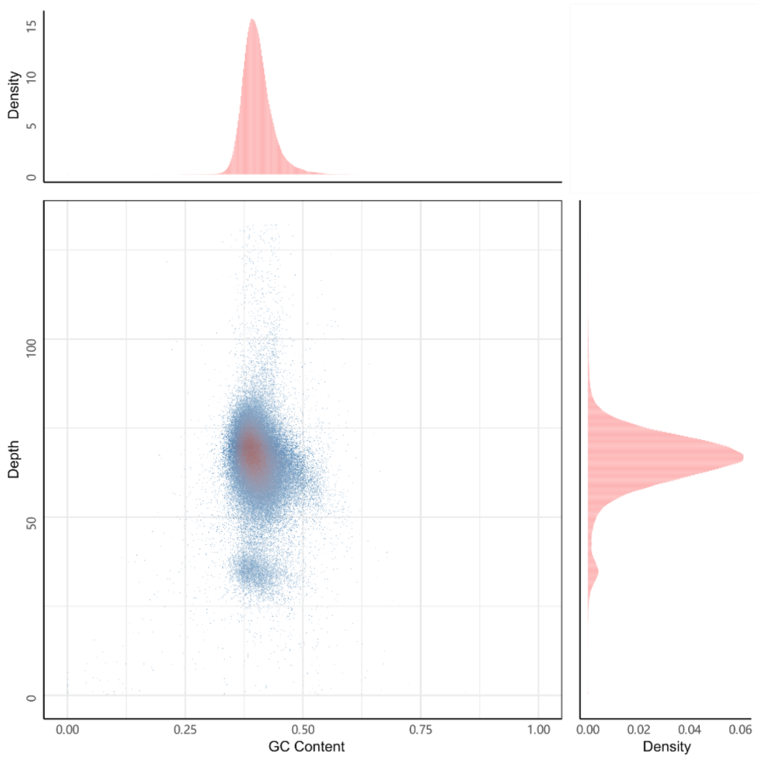

Fig. S3. Distribution map for GC content analysis of *M. electricus*. The horizontal coordinates indicate the GC content and the vertical coordinates indicate the sequencing depth. On the right is the contig coverage depth distribution and on the top is the GC content distribution. It can be seen that in the part of the red area, the GC distribution of this species shows a major regional distribution, centered around depth 70, corresponding to the contig coverage distribution on the right, with a pure peak at position 70; corresponding to the GC content distribution above, the main peak is at the GC content of 40%, which is basically consistent with the GC content of the genome we calculated (40.45%), and the red scatter is also distributed around, indicating that the genome is not contaminated by other exogenous species.

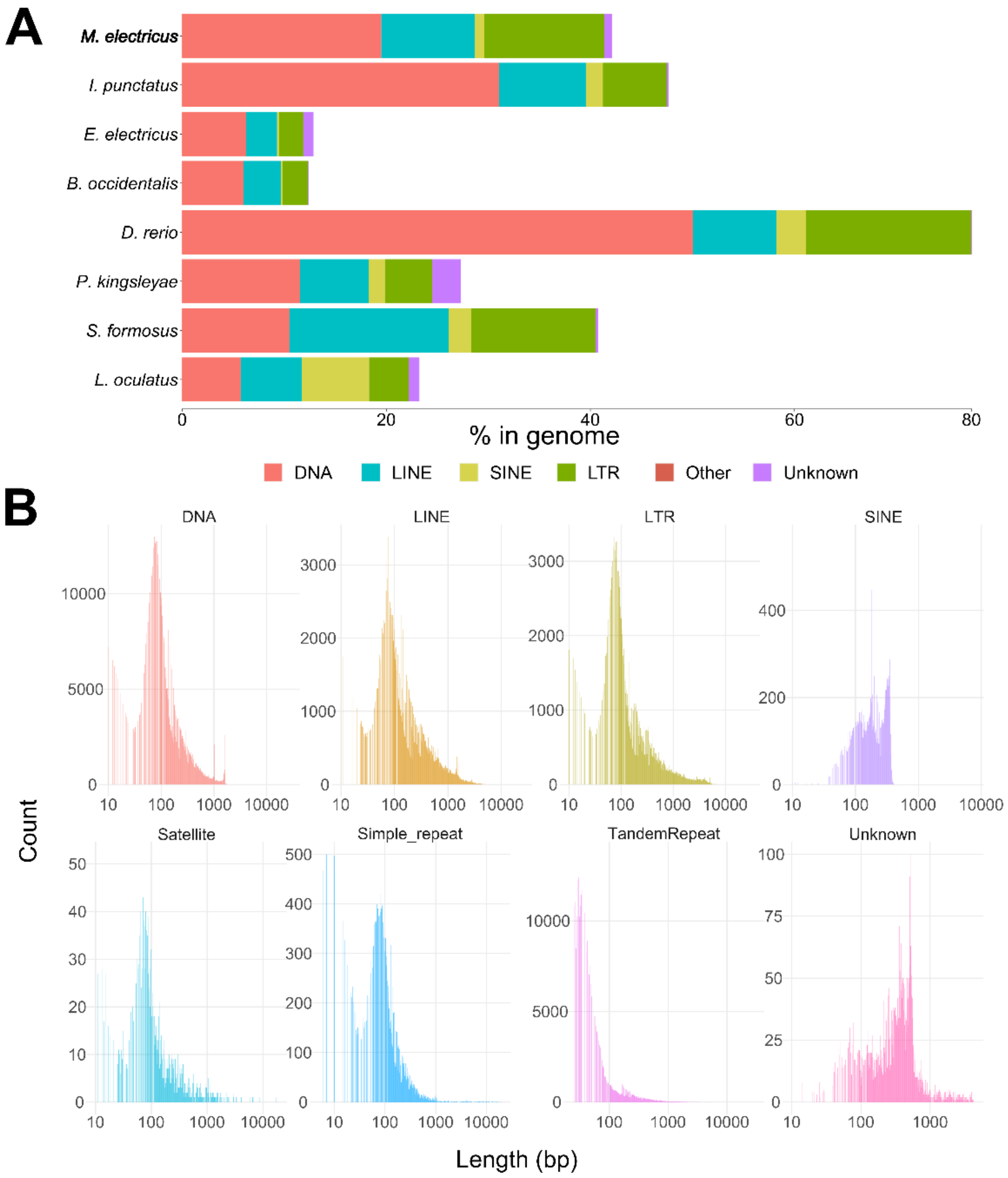

Fig. S4. Repeat annotation of *M. electricus* genome. A, the proportion of transposable elements (TEs) in fish genomes (*M. electricus*, *I. punctatus*, *E. electricus*, *B. occidentalis*, *D. rerio*, *P. kingsleyae*, *S. formosus* and *L. oculatus*). B, the distribution of length of major types of TEs.

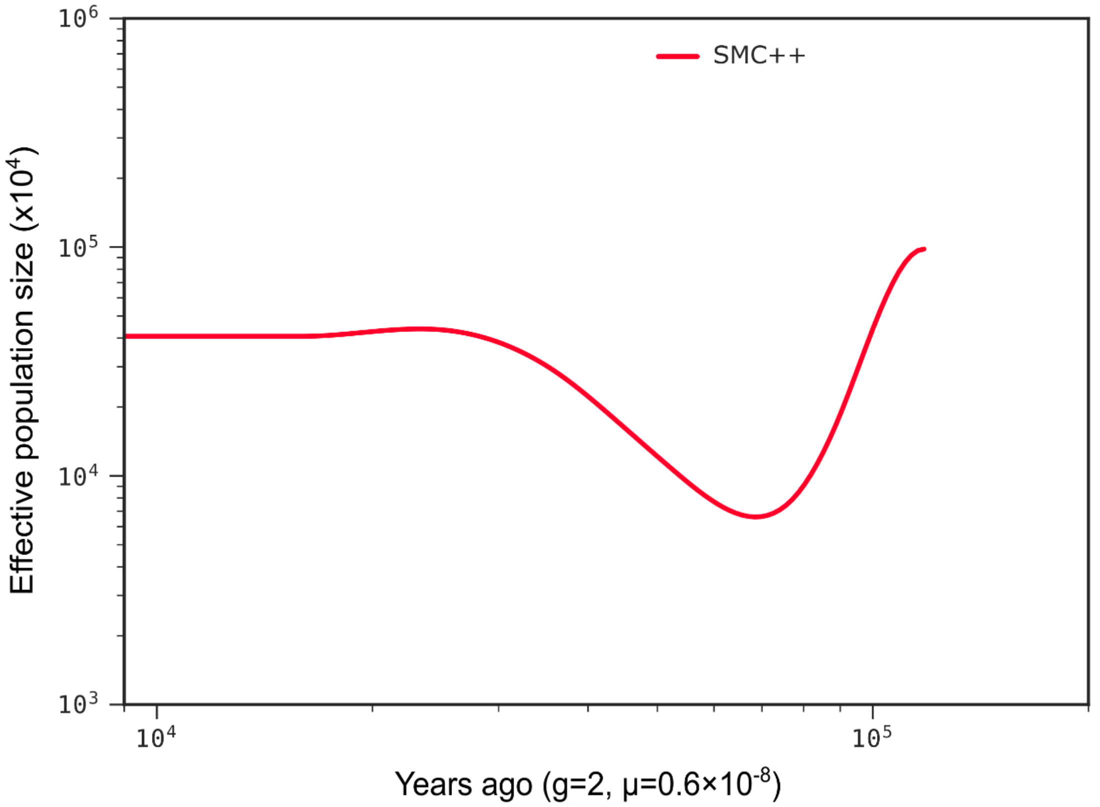

Fig. S5. Demographic history of *M. electricus* inferred by SMC++ using mutation rate of 6×10^{-9}.

Supplementary Tables

Table S1. Sequenced data statistics.

Category	Bases number(bp)	genome coverage	read length
stLFR	118,870,983,600	~82 ×	PE 100+140
Hi-C	307,073,045,400	~211 ×	PE 100+100

Table S2. Assembly statistics.

Category	Total Scaffold number	Scaffold Length	Scaffold N50	Scaffold N90	Contig Number	Contig Length	Contig N50	Contig max length	GC (%)
stLFR	17,528	798,763,704	3,588,439	42,546	79,417	735,063,284	18,281	4,492	40.45
Gapcloser	17,491	796,449,416	3,578,488	42,201	39,158	740,302,697	48,402	9,221	40.45

Table S3. Reference genomes and annotations used for gene prediction and comparative genomic analysis.

Common Name	Scientific Name	RefSeq Assembly	Genome and/or Annotation Release link	Date	Annot. Release
black bullhead catfish	*Ameiurus melas*	AMELA_1.0 (GCA_012411365.1)	https://ftp.ncbi.nlm.nih.gov/genomes/all/GCA/012/411/365/GCA_012411365.1_AMELA_1.0/	2020/4/16	
elephantfish	*Brachyhypopomus occidentalis*	ASM2036802v1 (GCA_020368025.1)	https://ftp.ncbi.nlm.nih.gov/genomes/all/GCA/020/368/025/GCA_020368025.1_ASM2036802v1/		
zebrafish	*Danio rerio*	GRCz11 (GCA_000002035.6)	https://ftp.ncbi.nlm.nih.gov/genomes/all/annotation_releases/7955/106/GCF_000002035.6_GRCz11/	2017/6/2	106
electric Eel	*Electrophorus electricus*	fEleEle1.pri (GCA_013358815.1)	https://ftp.ncbi.nlm.nih.gov/genomes/all/annotation_releases/8005/101/GCF_013358815.1_fEleEle1.pri/	2020/7/10	101
Asian red tailed catfish	*Hemibagrus wyckioides*	SWU_Hwy_1.0 (GCA_019097595.1)	https://ftp.ncbi.nlm.nih.gov/genomes/all/GCA/019/097/595/GCA_019097595.1_SWU_Hwy_1.0/	2021/7/6	
channel catfish	*Ictalurus punctatus*	IpCoco_1.2 (GCA_001660625.1)	https://ftp.ncbi.nlm.nih.gov/genomes/all/annotation_releases/7998/100/GCF_001660625.1_IpCoco_1.2/	2016/7/1	100
spotted gar	*Lepisosteus oculatus*	LepOcu1 (GCA_000242695.1)	https://ftp.ncbi.nlm.nih.gov/genomes/all/annotation_releases/7918/101/GCF_000242695.1_LepOcu1/	2016/1/6	101
striped catfish	*Pangasianodon hypophthalmus*	GENO_Phyp_1.0 (GCA_009078355.1)	https://ftp.ncbi.nlm.nih.gov/genomes/all/GCA/009/078/355/GCA_009078355.1_GENO_Phyp_1.0/	2019/10/22	101
elephantfish	*Paramormyrops kingsleyae*	PKINGS_0.1 (GCA_002872115.1)	https://ftp.ncbi.nlm.nih.gov/genomes/all/annotation_releases/1676925/100/GCF_002872115.1_PKINGS_0.1/	2018/1/31	100
whale shark	*Rhincodon typus*	ASM164234v2 (GCF_001642345.1)	https://ftp.ncbi.nlm.nih.gov/genomes/all/annotation_releases/259920/100/GCF_001642345.1_ASM164234v2/	2017/3/13	100
Asian bonytongue	*Scleropages formosus*	fSclFor1.1 (GCA_900964775.1)	https://ftp.ncbi.nlm.nih.gov/genomes/all/annotation_releases/113540/101/GCF_900964775.1_fSclFor1.1/	2019/5/6	101
Chinese large-mouth catfish	*Silurus meridionalis*	ASM1480568v1 (GCA_014805685.1)	https://ftp.ncbi.nlm.nih.gov/genomes/all/GCA/014/805/685/GCA_014805685.1_ASM1480568v1/	2020/10/2	100
fugu	*Takifugu rubripes*	fTakRub1.2 (GCA_901000725.2)	https://ftp.ncbi.nlm.nih.gov/genomes/all/annotation_releases/31033/103/GCF_901000725.2_fTakRub1.2/	2019/6/20	103

Table S4. The SRA data information used for this study.

Species	Tissue	SRA accession NO.	Raw reads	Clean reads
Malapterurus electricus	electric organ	SRR1299090<br>SRR1299753<br>SRR1299754	19,959,132,300	12,530,968,200
	skeletal muscle	SRR1299091	5,165,014,500	2,908,391,100

Table S5. UniProt accession ID of taste receptor (TR) genes.

gene	UniProt ID	name	species
pkd2l1	A0A4W4ESW4	Dare_pkd2l1	*Oryzias. latipes*
	A0A8J4TTH4	Clma_pkd2l1	*Electrophorus. electricus*
	pkd2l1-201	Elel_pkd2l1	*Clarias. magur*
	B0V3C8	Atsp_Pkd2l1	*Atractosteus spatula*
tas1r1	A4PHQ6	Dare_tas1r1-3	*Danio. rerio*
	Q2MHL2	Dare_tas1r1-2	*Danio. rerio*
	Q2MHK6	Dare_tas1r1-1	*Danio. rerio*
	A4PHQ7	Orla_tas1r1	*Oryzias. latipes*
tas1r2	A4PHQ8	Dare_tas1r2.1	*Danio. rerio*
	Q2MHK5	Dare_tas1r2.2	*Danio. rerio*
	Q2MHK4	Orla_tas1r2a	*Oryzias. latipes*
	Q2MHK3	Orla_tas1r2b	*Oryzias. latipes*
	Q2MHK9	Orla_tas1r2c	*Oryzias. latipes*
tas1r3	A0A8B9H395	Dare_tas1r3	*Danio. rerio*
	A0A8C4S4T8	Asme_tas1r3	*Astyanax mexicanus*
	H2LH38	Erca_tas1r3	*Erpetoichthys. calabaricus*
	A0A2D0R6Q1	Orla_tas1r3	*Oryzias. latipes*
tas2r	A0A7N9AMK6	Icpu_tas2r	*Ictalurus punctatus*
	Q2MHK8	Maar_tas2r	*Mastacembelus armatus*
	Q2MHK7	Dare_tas2r200.1	*Danio. rerio*
	Q58WW8	Dare_tas2r200.2	*Danio. rerio*
	A6P6V6	Dare_tas2r203	*Danio. rerio*
	Q2AB30	Dare_tas2r202	*Danio. rerio*
	A6P6V3	Dare_tas2r201	*Danio. rerio*

Table S6. Summary statistics of scaffolds-level genome assembly of the *M. electricus*.

	Contig		Scaffold	
	Size (bp)	Number	Size (bp)	Number
N90	9,221	17,381	42,201	494
N80	18,249	11,748	820,167	189
N70	27,610	8,476	1,848,505	122
N60	37,515	6,180	2,781,030	86
N50	48,402	4,445	3,578,488	61
Longest	407,165		14,448,789	
Total Size	740,290,815		796,449,416	
Total Number (>100 bp)	38,960		17,491	
Total Number (>2 kb)	31,008		12,562	
GC content	40.45%		40.45%	
Ratio of Ns			56,146,719 (7.05%)	

Table S7. The length and statistics of contigs and gaps for chromosomes.

Chromosome	Length (Mb)	Contig number	N base (Mb)	Gap number	Max contig length (Mb)	Min contig length (kb)	Contig n50 (kb)
chr1	52.40	1371	3.71	1370	0.34	0.51	54.79
chr2	36.05	997	2.95	996	0.28	0.53	49.36
chr3	33.82	890	2.42	889	0.33	0.56	55.51
chr4	33.45	863	2.49	862	0.30	0.53	55.19
chr5	32.71	868	2.49	867	0.33	0.52	54.96
chr6	29.94	831	2.52	830	0.25	0.51	52.22
chr7	29.34	807	2.22	806	0.25	0.54	55.05
chr8	29.00	822	2.23	821	0.21	0.51	50.01
chr9	27.70	708	1.72	707	0.41	0.82	60.53
chr10	26.81	652	1.99	651	0.23	0.55	59.87
chr11	26.06	667	1.87	666	0.35	0.61	59.39
chr12	25.63	630	1.93	629	0.36	0.54	59.94
chr13	25.46	691	1.75	690	0.24	0.56	54.17
chr14	25.39	696	1.85	695	0.36	0.53	55.95
chr15	24.18	637	1.85	636	0.26	0.56	55.12
chr16	23.75	588	1.58	587	0.25	0.57	54.83
chr17	23.49	631	1.49	630	0.18	0.56	52.81
chr18	22.48	614	1.71	613	0.18	0.54	56.06
chr19	22.03	612	2.03	611	0.24	0.64	51.37
chr20	20.48	556	1.60	555	0.23	0.52	55.89
chr21	19.89	505	1.57	504	0.26	0.50	55.76
chr22	19.79	518	1.40	517	0.20	0.60	57.00
chr23	18.52	499	1.57	498	0.27	0.54	57.45
chr24	18.44	512	1.62	511	0.24	0.50	52.81
chr25	17.37	467	1.54	466	0.26	0.52	55.68
chr26	17.30	473	1.31	472	0.23	0.54	54.16
chr27	14.26	421	1.52	420	0.21	0.69	47.86
chr28	12.99	361	0.99	360	0.32	0.62	47.51
unanchored	88.05	20274			0.19	0.50	6.01

Table S8. Summary of BUSCO notation assessment of the *M. electricus* assembly.

Database	Complete BUSCOs (C)	Complete and single-copy BUSCOs (S)	Complete and duplicated BUSCOs (D)	Fragmented BUSCOs (F)	Missing BUSCOs (M)	Total BUSCO groups searched
actinopterygii (*obd10*)	3173(87.2%)	3083(84.7%)	90(2.5%)	142(3.9%)	325(8.9%)	3640
metazoa (*odb10*)	871(91.3%)	834(87.4%)	37(3.9%)	27(2.83%)	56(5.9%)	954
vertebrata (*odb10*)	2833(84.4%)	2778(82.8%)	55(1.6%)	268(7.99%)	253(7.6%)	3354

Table S9. Heterozygosity rate estimation based on of k-mer analysis.

k-mer size	17	15
Total k-mer number	49,830,038,647	51,533,475,112
Raw peak depth	60	62
Estimated genome size	831,185,082 bp	830,500,644 bp
Ratio of kmer-species heterozygous	0.0317	0.0081
Rate of heterozygosity (%)	0.19	0.05

Table S10. The accuracy estimation of genome assembly for *M. electricus* based on SNP calling.

SNP	Number	Percentage of SNP (%)	Percentage of genome (%)	Percentage of genome without gaps (%)
All SNP	348,615	100	0.044	0.047
Heterozygosis SNP	344,204	98.73	0.043	0.043
Homology SNP (assembly error ratio)	4,411	1.27	0.00055	0.00055
Homology SNP in CDS	94	2.13		
Homology SNP in non-coding DNA	1,072	24.30		
Homology SNP in intergenic region	3,245	73.57		

Table S11. Summary statistics of interspersed repeats in the of the *M. electricus* genome.

Type	Repbase TEs		TE proteins		*de novo*		Combined TEs	
	Length (bp)	% in genome	Length (bp)	% in genome	Length (bp)	% in genome	Length (bp)	% in genome
DNA	82,418,272	10.35	23,499,679	2.95	128,015,090	16.07	155,437,673	19.52
LINE	27,704,821	3.48	21,462,046	2.69	58,511,305	7.35	72,877,212	9.15
SINE	7,299,752	0.92	0	0	343,188	0.04	7,513,824	0.94
LTR	21,812,826	2.74	17,149,190	2.15	84,024,926	10.55	93,686,601	11.76
Other	14,923	0.002	0	0	0	0	14,923	0.002
Unknown	0	0	0	0	5,796,366	0.73	5,796,366	0.73
Total	126,374,570	15.87	62,065,438	7.79	246,787,206	30.99	264,061,593	33.15

Table S12. Major types of transposable elements (TEs) in the *M. electricus* genome.

Table S13. Summary statistics of predicted protein-coding genes in the *M. electricus* genome.

Method	Gene set	Total Genes Predicted	Average Gene Length (bp)	Average CDS Length (bp)	Average Exons per Gene	Average Exon Length (bp)	Average Intron Length (bp)
de novo	Augustus	36,423	9,053.06	1,316.08	5.43	242.55	1,748.07
Homolog	actinopterygii_odb9	4,828	11,920.46	1,485.24	9.20	161.42	1,272.45
	Danio rerio	17,420	14,569.43	1,622.60	9.16	177.09	1,586.09
	Electrophorus electricus	18,563	15,629.43	1,739.45	9.80	177.58	1,579.22
	Ictalurus punctatus	21,085	15,555.46	1,724.71	9.67	178.35	1,595.14
	Lepisosteus oculatus	14,384	15,237.25	1,602.85	9.30	172.33	1,642.45
	Rhincodon typus	12,434	9,904.94	1,205.37	7.28	165.51	1,384.72
	Takifugu rubripes	14,268	14,697.71	1,610.99	9.42	171.01	1,554.15
RNAseq	cufflinks	23,130	9,404.67	930.48	6.16	151.08	1,415.55
Integration	GeMoMa	20,093	16,853.33	1,627.05	9.09	178.94	1,791.33
Final		19,985	16,149.91	1,629.58	9.06	179.77	1,800.49

Table S14. Comparison of the *M. electricus* genome annotation with those of four other related fishes.

	Ostariophysi				
	Cypriniformes	Gymnotiformes	Siluriformes		
	Danio rerio	*Electrophorus electricus*	*Ictalurus punctatus*	*Pangasianodon hypophthalmus*	*Malapterurus electricus*
Number of genes	28,428	22,839	22,266	22,681	19,985
Average gene length (bp)	26,741.00	17,729.86	12,936.01	17,328.98	16,149.91
Average CDS length (bp)	1,749.89	1,762.56	1,821.60	1,801.95	1,629.58
Average exon length (bp)	182.79	170.86	169.93	170.21	179.77
Average intron length (bp)	2,915.05	1,713.97	1,143.47	1,619.62	1,800.49

Table S15. Number of predicted genes in *M. electricus* genome functionally annotated using indicated databases.

Database	Number	Percent (%)
Swissprot	18,100	90.57%
KEGG	17,183	85.98%
TrEMBL	18,937	94.76%
Interpro	18,285	91.49%
Overall	19,011	95.13%
Total Gene	19,985	

Table S16. Shared homologous synteny blocks (HSBs) in 6 Siluriformes species.

Species	Homologous block length (bp)	Chromosome -level genome length (bp)	Coverage (%)
Malapterurus electricus	242,534,038	708,703,171	34.22
Pangasianodon hypophthalmus	238,485,665	752,696,875	31.68
Hemibagrus wyckioides	234,691,772	771,597,054	30.42
Ameiurus melas	262,524,731	855,611,017	30.68
Silurus meridionalis	233,746,698	738,971,436	31.63
Ictalurus punctatus	250,285,008	762,005,527	32.85

Table S17. Gene family cluster results of *M. electricus*, *E. electricus*, *P. kingsleyae*, *P. hypophthalmus*, *I. punctatus*, *D. rerio*, *S. formosus* and *L. oculatus*.

Species	Genes number	Genes in families	Unclustered genes	Family number	Unique families	Average genes per family
I. punctatus	22,367	21,608	759	15,347	81	1.40
P. hypophthalmus	22,487	21,975	512	15,567	52	1.41
M. electricus	19,399	17,281	2,118	12,996	139	1.32
E. electricus	21,899	21,344	555	14,961	73	1.42
D. rerio	27,979	26,283	1,696	15,292	309	1.71
P. kingsleyae	23,585	21,780	1,805	14,568	82	1.49
S. formosus	23,371	22,198	1,173	14,490	57	1.53
L. oculatus	18,526	16,997	1,529	13,497	94	1.25

Table S18. The gene ontology (GO) enrichment results of 139 unique gene families (including 384 genes) from *M. electricus*.

Table S19. The GO enrichment results in biological process for genes from 295 expanded gene families (including 708 genes) in *M. electricus* genome.

Table S20. Positively selected genes (PSGs) in *M. electricus*.

Table S21. Comparisons between the *M. electricus* genome and assembled genomes of other Siluriformes species.

Species	Family	Assembly size (Mb)	Number of chrs	Contig N50	Scaffold N50 (Mb)	GC (%)	Identified genes	Annotated genes	Repetitive elements (%)	Sources
Malapterurus electricus	Malapteruridae	796.5	28	48.4 kb	3.58	40.45	–	19,985	33.2	This study
Ameiurus melas	Ictaluridae	868.5	29	7.4 Mb	32.28	40.09	29,840	24,354	–	GenBank GCA_012411365.1
Bagarius yarrelli	Sisoridae	570.8	–	1.8 Mb	3.12	38	17,182	17,182	35.3	(Jiang et al. 2019)
Clarias magur	Clariidae	941.3	–	1.2 Mb	1.31	39.8	23,712	23,712	43.7	(Kushwaha et al. 2021)
Clarias batrachus	Clariidae	821	–	24.9 kb	361.1 kb	39.2	22,914	22,587	30.3	(Li et al. 2018)
Glyptosternon maculatum	Sisoridae	704.8	24	27.8 Mb	91.7	39.6	22,066	20,234	32.8	(Xiao et al. 2021)
Hemibagrus wyckioides	Bagridae	789.8	29	21.7 Mb	26.01	39.7	22,794	22,794	40.1	(Shao et al. 2021)
Ictalurus punctatus	Ictaluridae	821.8	29	81.4 kb	7.9	39.7	29,044	23,920	32.6	(Chen et al. 2016; Liu et al. 2016)
Ictalurus furcatus	Ictaluridae	825.5	29	16.8 Mb	30.4	39.5	–	33,686	47	(Wang et al. 2022)
Leiocassis longirostris	Bagridae	703.2	26	28.03 Mb	97.44	39.67	23,708	23,170	34	(He et al. 2021)
Pangasianodon gigas	Pangasiidae	827.2	30	40.5 kb	25.95	38.8	27,065	22,017	–	GenBank GCA_022758105.1
Pangasianodon hypophthalmus	Pangasiidae	759	30	1.6 Mb	26.46	38.3	25,113	21,247	33.83	(Kim et al. 2018)
Pelteobagrus fulvidraco	Bagridae	732.8	26	25.8 Mb	94.2	39.6	24,552	24,552	38.5	(Gong et al. 2018)
Pseudobagrus ussuriensis	Bagridae	742	26	28.53 Mb	97.34	39.33	24,075	22,039	40	(Zhu et al. 2022)
Pangasius djambal	Pangasiidae	844.5	30	28.8 kb	24.95	39.4	27,409	22,470	–	GenBank GCA_022985145.1
Silurus asotus	Siluridae	744.1	29	28.82 Mb	94	–	22,894	–	29.8	(Chen et al. 2021)
Silurus glanis	Siluridae	793.4	–	3.2 Mb	84.2	39.2	21,316	20,532	39.5	(Ozerov et al. 2020)
Silurus meridionalis	Siluridae	742.6	29	13.2 Mb	28.05	39	–	22,965	40.1	(Zheng et al. 2021)
Tachysurus fulvidraco	Bagridae	712	26	3.3 Mb	27.16	39.5	33,922	23,998	43.31	(Gong et al. 2018)

References in supplement materials

Chen W, Zou M, Li Y, et al (2021) Sequencing an F1 hybrid of Silurus asotus and S. meridionalis enabled the assembly of high-quality parental genomes. Sci Rep 11:13797. https://doi.org/10.1038/s41598-021-93257-x

Chen X, Zhong L, Bian C, et al (2016) High-quality genome assembly of channel catfish, Ictalurus punctatus. Gigascience 5:2–5. https://doi.org/10.1186/s13742-016-0142-5

Gong G, Dan C, Xiao S, et al (2018) Chromosomal-level assembly of yellow catfish genome using third-generation DNA sequencing and Hi-C analysis. Gigascience 7:1–9. https://doi.org/10.1093/gigascience/giy120

He W-P, Zhou J, Li Z, et al (2021) Chromosome-level genome assembly of the Chinese longsnout catfish Leiocassis longirostris. Zool. Res. 42:417–422

Jiang W, Lv Y, Cheng L, et al (2019) Whole-Genome Sequencing of the Giant Devil Catfish, Bagarius yarrelli. Genome Biol Evol 11:2071–2077. https://doi.org/10.1093/GBE/EVZ143

Kim OTP, Nguyen PT, Shoguchi E, et al (2018) A draft genome of the striped catfish, Pangasianodon hypophthalmus, for comparative analysis of genes relevant to development and a resource for aquaculture improvement. BMC Genomics 19:733. https://doi.org/10.1186/s12864-018-5079-x

Kushwaha B, Pandey M, Das P, et al (2021) The genome of walking catfish Clarias magur (Hamilton, 1822) unveils the genetic basis that may have facilitated the development of environmental and terrestrial adaptation systems in air-breathing catfishes. DNA Res 28:1–16. https://doi.org/10.1093/dnares/dsaa031

Li N, Bao L, Zhou T, et al (2018) Genome sequence of walking catfish (Clarias batrachus) provides insights into terrestrial adaptation. BMC Genomics 19:952. https://doi.org/10.1186/s12864-018-5355-9

Liu Z, Liu S, Yao J, et al (2016) The channel catfish genome sequence provides insights into the evolution of scale formation in teleosts. Nat Commun 7:1–13. https://doi.org/10.1038/ncomms11757

Ozerov MY, Flajšhans M, Noreikiene K, et al (2020) Draft genome assembly of the freshwater apex predator wels catfish (Silurus glanis) using linked-read sequencing. G3 Genes, Genomes, Genet 10:3897–3906. https://doi.org/10.1534/g3.120.401711

Shao F, Pan H, Li P, et al (2021) Chromosome-Level Genome Assembly of the Asian Red-Tail Catfish (Hemibagrus wyckioides). Front Genet 12:747684. https://doi.org/10.3389/fgene.2021.747684

Wang H, Su B, Butts IAE, et al (2022) Chromosome-level assembly and annotation of the blue catfish Ictalurus furcatus, an aquaculture species for hybrid catfish reproduction, epigenetics, and heterosis studies. Gigascience 11:. https://doi.org/10.1093/gigascience/giac070

Xiao S-J, Mou Z-B, Yang R-B, et al (2021) Genome and population evolution and environmental adaptation of Glyptosternon maculatum on the Qinghai-Tibet Plateau. Zool Res 42:502–513. https://doi.org/10.24272/j.issn.2095-8137.2021.096

Zheng S, Shao F, Tao W, et al (2021) Chromosome-level assembly of southern catfish (silurus meridionalis) provides insights into visual adaptation to nocturnal and benthic lifestyles. Mol Ecol Resour 21:1575–1592. https://doi.org/10.1111/1755-0998.13338

Zhu C, Liu H, Pan Z, et al (2022) Insights into chromosomal evolution and sex determination of Pseudobagrus ussuriensis (Bagridae, Siluriformes) based on a chromosome-level genome. DNA Res 29:. https://doi.org/10.1093/dnares/dsac028
